# Undertaking a high stakes virtual OSCE (“VOSCE”) during Covid-19

**DOI:** 10.1186/s12909-021-02660-5

**Published:** 2021-04-20

**Authors:** Jenny Blythe, Nimesh S. A. Patel, Will Spiring, Graham Easton, Dason Evans, Egle Meskevicius-Sadler, Hassan Noshib, Heather Gordon

**Affiliations:** Queen Mary University of London, Barts and The London School of Medicine & Dentistry, Institute of Health Sciences Education, Centre for Medical Education, Whitechapel, London, E1 1AS UK

**Keywords:** OSCE, Assessment, Virtual, Remote, Medical students

## Abstract

**Background:**

The Covid pandemic and associated lockdown forced medical schools globally not only to deliver emergency remote teaching, but to consider alternative methods of high stakes assessment. Here we outline our approach to the resit virtual OSCE (“VOSCE”) for final year medical students that we undertook during “lockdown” in the current pandemic.

**Methods:**

The original ‘pre Covid’ examination blueprint was reviewed and modified for the virtual environment in both format and content. In anticipation of the new format delivery, a number of pre-training sessions took place for all parties, and standardised templates were developed.

**Results:**

A total of 9 students undertook the VOSCE, which took the form of a two-part exam (a communication and clinical examination component, and a practical procedures component). The VOSCE was completed by all students, examiners, simulated patients and invigilators on an online digital platform with no issues with regards to technical problems.

**Conclusions:**

A total of 6 students passed the VOSCE and as such progressed to graduation. The limitation of assessing some particular types of skills across the remote format (such as practical procedures) was recognised. The training and the templates developed were helpful in case the VOSCE format needs to be adopted in future at short notice and/or expanded in future.

## Background

The Covid pandemic and associated lockdown forced medical schools globally not only to deliver emergency remote teaching, but to consider how alternative methods of high stakes assessment should go ahead. Here we outline our approach to a resit virtual OSCE (“VOSCE”) for Final year medical students that we undertook during “lockdown” in the current pandemic.

Objective Structured Clinical Examinations (OSCEs) were first described by Harden in 1975, and their use has become widespread in the field of undergraduate and postgraduate education [1, 2]. The OSCE was first designed to introduce standardisation and reduce the number of variables that could impact the assessment of performance. Hence, in a well-designed OSCE, the grades of the candidates should be determined by the performance of the candidates, with minimal effect from other sources of variance [2].

Using virtual technology to assess students involves a wide range of formats, and the technology used can be complex or straightforward, although in simulation it is recognised that a high-tech ‘realistic’ reproduction of the environment, sometimes called high-fidelity, is less important than suspension of disbelief by the participants ‘in the moment’ [3].

The literature around ‘virtual’ or ‘remote’ OSCEs is limited in comparison to that of OSCEs held in the traditional format. In 2008, Courteille and colleagues ran a one virtual OSCE station using a platform designed to assist clinical healthcare students to explore and solve a clinical case by navigating a virtual patient’s history (through texts and videos), video of examinations and data interpretation [4]. More recently, a virtual OSCE pilot preparing trainees for telehealth has also been reported [5]. Virtual OSCEs have also been recognised as effective for both learning and evaluating clinical competence in Nurse Practitioner students [6].

During the global Covid-19 pandemic, the first-sit OSCE for our Final Year medical students fell just before official first ‘lockdown’ was declared in the UK, and needed to be modified at short notice to abide by extra hygiene and social distancing. A similar social distancing OSCE has been described elsewhere [7]. However, at the start of lockdown, for our second-sit OSCE, calendared for ten weeks after the first sitting, it was decided to plan for an entirely distanced delivered OSCE.

Currently all UK medical schools plan their own curriculum, including where finals exams fall scholastically, and as such each medical school had to make a judgement about whether to continue with finals in the light of the pandemic or use other evidence of achievement to progress final year students to become doctors. We decided to pursue a VOSCE rather than use alternative methods of student assessment for various reasons: firstly, we wanted parity of assessment between the two sittings of students, secondly, we recognised we had time to plan and develop a robust assessment and were confident that we could use the technology we were already familiar with as remote working educators.

## Methods

### Planning stage

In usual circumstances, the second-sit OSCE, run for those students who either failed or did not sit the first attempt, is made up from “first sit” OSCE stations from previous years. The justification for this is that a pass-mark using the Borderline Regression Method (BRM) for a similar cohort has already been generated for this exam, a standard setting method that would not be feasible for the small cohort that take the second-sit.

Our Final Year OSCE usually comprises of three parts- a five-station physical examination component (using real patients in the hospital setting), a six-station communication skills component (using trained Simulated Patient actors) and a six-station practical procedures component (which on occasion uses an unscripted patient eg blood pressure). In all three exam components, follow-up questions are asked after students have demonstrated their examination, consultation or practical procure skills, in order to assess their clinical reasoning. We had already had to modify the first-sit OSCE in order that real patients were not involved and more physical distancing was present between stations, so our first-sit OSCE this year had comprised of one examination station (a mental health assessment) and four communication stations in one component and six practical procedures stations in a second component.

To plan for the VOSCE, the second-sit blueprint that had been planned pre-Covid was initially reviewed by a group of academics involved in both assessment and delivery of the Final Year curriculum. The first decision was to select the stations from that blueprint to use in order to have the same balance of station components as the first sit, and then modify those stations in order that they would work in the virtual format.

In planning for the VOSCE, we acknowledged that:

1. Although there are specific skills and considerations around conducting video-consultations with patients [8, 9], these had not been covered in our students’ curriculum, and as such could not be assessed.
2. The examination and practical procedure stations would need to be a mix of students actively demonstrating examination skills (eg asking patients to stick their tongue out or demonstrate eye movements over video) and talking through elements not possible to replicate remotely (stating what you would be looking for on fundoscopy).
3. Some of our students taking the exam were based outside the UK and as such that influenced the equipment and paperwork that could be sent to them, and the internet based supporting resources (such as access to online drug formularies) that we could expect students to use during the exam.
4. Online platform(s) available to support delivery needed to be widely available to all parties involved (students, academics, examiners, support staff and simulated patients) with training and back-up support available where required.
5. As a new format of delivery, students and examiners would be too busy on what they were required to do and so we decided each student would need to be chaperoned by an invigilator. The invigilator would keep the timings and act as observer and trouble shooter as required.
6. We had no previous templates or guidance to work from in terms of planning, training or delivery so this would be an iterative process in terms of development.

Further planning activities were undertaken:

i) Weekly progress meetings with academics in terms of design of stations, with external examiners informed regarding decisions at each step.
ii) A mock VOSCE was designed to familiarise students with the exam format and technology, and for assessment support staff to practice logistics and identify potential problems with regards to running the VOSCE. All students attended this session using video conferencing software.
iii) Paperwork for several stations would be required by students during the exam such as patient notes or a drug chart. As we wanted to keep as much of the second-sit OSCE the same as the first-sit we decided to send physical paperwork to students in the post. To avoid any one student gaining an advantage the paperwork for each station was placed in its own envelope and sealed with security stickers. Students were instructed not to open the envelope until instructed by an invigilator on the day of the exam.
iv) Simulated patients selected for the VOSCE had undertaken the same communication skills stations previously in traditional format OSCEs, and so were familiar with station format and content.
v) Specific training was given to simulated patients and examiners to ensure full familiarity with the technology prior to the date of the VOSCE.

All these training sessions were recorded to allow those that could not attend the opportunity to watch the briefing and also allow those that attended the opportunity to review the briefing.

### Delivery stage

A standardised examiner and student briefing was undertaken, and an overall spreadsheet was designed that outlined timings for the exam (see Table 1). Examiners and simulated patients were sent bespoke spreadsheets with their timings. Delivery was via the MS Teams platform. Students were allocated a single invigilator from the Assessment Unit that guided them through the exam stations and who was responsible for “calling in” both examiner and simulated patients (if latter required) into the ‘room’ to examine. Individual student spreadsheets were also designed (see Table 2). Each student had their own MS Teams meeting room space and the link was distributed to examiners and simulated patients as necessary.

**Table 1:** Overall Master Plan of VOSCE

Part 1: Session 2 11.00–12.33: 5 Students								
Station no	Station Title	Simulated Pt	Examiner	START TIMES FOR STUDENTS AT EACH STATION				
				Student i	Student ii	Student iii	Student iv	Student v
Station 1	Hx of loss of consiousness	a	A	11:10:00	12:18:00	12:01:00	11:44:00	11:27:00
Station 2	Chronic disease management	b	B	11:27:00	11:10:00	12:18:00	12:01:00	11:44:00
Station 3	Cranial nerve examination	c	C	11:44:00	11:27:00	11:10:00	12:18:00	12:01:00
Station 4	Explanation of new medication	d	D	12:01:00	11:44:00	11:27:00	11:10:00	12:18:00
Station 5	Telephone referral	n/a	E	12:18:00	12:01:00	11:44:00	11:27:00	11:10:00

**Table 2:** Example of an individual student candidate VOSCE plan

Student iv							
Examiner	Simulated Pt	Station No and Title	Station Start & Finish time		Changeover	Station Notes	Tag envelope
D	d	4. Explanation of new medication	11:10:00	11:25:00	11:27:00	needs BNF	
E	n/a	5. Telephone referral	11:27:00	11:42:00	11:44:00	1 x Envelope	63,073
A	a	1. History Taking-Loss of Consciousness	11:44:00	11:59:00	12:01:00		
B	b	2. Chronic disease management	12:01:00	12:16:00	12:18:00		
C	c	3. Cranial nerve examination	12:18:00	12:33:00	**End of exam**		

**Invigilator Instructions:**

Wait for confirmation that central briefing is over before calling Student

**Station 4**

11:00 Call Student

Commence briefing-ask Student to open BNF website

11:09 Call Examiner (A) and Simulated Patient (a) into the room: Introduce Examiner and patient; Please confirm that you can see and hear each other well

11:10 Share the screen with Student Instructions, wait for Student to confirm ‘Yes’

11:10 Start the station audio file. Audio File: “Please start your preparation time”

11:15 Audio File: “That’s the end of the preparation time. Please start working on the station”

11:24 Audio File “You have one minute remaining”

11:25 Audio File: “That is the end of the station please stop working”

Invigilator:

i) Ask Student to close BNF website

ii) Say “We will have 2 min changeover time now”

iii) Stop sharing Student Instructions, locate the next station Student Instructions, locate the next audio guide

iv) Instruct Student “Please pick up Station 5 envelope and open it. Please do not start reading until we start the station”

v) Ensure exit of Examiner (A) and Simulated Patient (a)

**Station 5** Call Examiner (B) and Simulated Patient (b) into the room:

Introduce Examiner; Please confirm that you can hear each other well

Share the screen with Student Instructions, wait for Student to confirm ‘Yes’

11:27 Start the station audio file. Audio File: “Please start your preparation time”

11:32 Audio File: “That’s the end of the preparation time. Please start working on the station”

Unlike our face to face OSCEs, students were given time before the VOSCE stations started, in order for all parties present to introduce themselves. The examiner, invigilator and any observers then switched off their camera so as not to distract the interaction between student and ‘patient’. At this point, the student was prompted to open any security sealed paperwork, or to open relevant websites (eg online drug formularies) for the VOSCE station purposes. Station instructions were displayed to the student via invigilators sharing their screen during the station preparation time to add a level of security to the examination documents.

Over the last 3 years, we have transitioned from scanned paper-based candidate mark-sheets to an electronic tablet-based making system for OSCEs. Neither of these approaches were feasible given the restrictions of lockdown and the various locations of examiners and other staff. After various trials of online forms and spreadsheets, it became clear that each examiner should use a local copy of mark sheets. The exams team produced dedicated Microsoft Excel workbooks for each individual examiner with separate candidate mark sheets presented in the order that the candidates would ‘arrive’ at their station. These mark sheets utilised cell locking, data validation, conditional formatting and logic formulae to highlight any missing marks and/or qualitative feedback. In this way many of the quality improvements that had been gained with the tablet-based system were maintained.

It was agreed to send the Excel workbook of marksheets individually to each examiner on the day before the VOSCE to ensure they had time to familiarise themselves with the marksheet and the examiner instructions. This is an unusual practice at our institution where familiarisation usually occurs on the day of the exam. This change was driven by the new format of the mark sheets, in addition to examiners wishing to trial how they could manage marking and observing using multiple screens or devices. Any perceived risk to exam security was mitigated by the small size of the assessment and the hand-picked nature of the examiners.

Some stations would normally require the candidate to complete and hand in a document for marking. One example would be a prescription station. For these stations, dedicated additional time was allocated for students to either show or photograph and email any relevant completed paperwork.

In addition, the exam applied the medical school policy to reasonably adjust for specific learning differences and provide students with extra time. Under this policy, reading time is explicitly set aside for all students and extended for those with examination access arrangements. This was accommodated using specific time settings and extended time by 25% on these stations.

After the exam, there was an immediate post-VOSCE feedback session for examiners, simulated patients, invigilators and academic staff so that any unforeseen challenges along with any problems could be captured.

### Standard setting

It was determined that the previous BRM standard set score could not be used for any individual station as the format of delivery had changed significantly. The standard was therefore set using the Angoff method, a consensus building technique, first described in a brief footnote in a text by William Angoff [10]. The Angoff panel comprised 8 academics, all involved in both clinical work and teaching, and all with a clear concept of the appropriate level of a newly qualified doctor. Members of the Angoff panel had experience in OSCEs ranging from 5 years to over 30 years. Those taking part in the Angoff were given guidance which provided an overall anchor statement of:” Imagine a FY1 (Foundation Year 1-a newly qualified doctor) on their first day who is just acceptably competent (minimally acceptable level of competence). What we are asking, is that out of 100 such FY1 at a just acceptable level, What proportion of minimally competent candidates at the level of an FY1 on day 1 will get the maximum score possible for this item?”

This item-based standard setting was initially done individually, and then all item data were combined and discussed at a consensus meeting. Considerable time (approx 3 h) was taken in this meeting justifying individual results, with detailed discussion where variance in the item score was greater than plus or minus 2 standard deviations from the mean for the item.

Given that the marksheet had checklist items with the following gradings of 1,0; 2,1,0; 5,2,0; and 7,5,0 with the mark sheets predominately having more 0,1 items the question “what score will the borderline candidate achieve?” meant that there would be artificial raising or lowering of the pass mark for a station, as standard setters would choose 1 or 0 this giving the station a high or low pass mark. The Angoff pass mark was compared with previous stations that had a borderline line regression pass mark and as they were compatible there was confidence with the process used.

Once the full Angoff process was completed, the panel were then informed of any previous passing standards of stations set through the BRM. Where there were significant differences, possible reasons for this were explored. Individual standard setting was conducted before the OSCE, however the consensus-building meeting occurred after the assessment had run. This was useful as it had become clear that the video format itself, rather than level of competence, resulted in some items being less likely to be demonstrated, handwashing being a rather obvious example. Individual scores of examiners were unknown at the consensus-building meeting.

Prior to the meeting the standard set across all stations averaged 63.48% and following the meeting the standard set was modified to 63.50%. Of note, the pass rate for the VOSCE was similar to previous OSCES although derived by an alternative method, which gave greater confidence in the standard setting process.

## Results

A total of 9 students undertook the VOSCE; 3 of these students were “first sit” (ie they did not attend the face to face OSCE that was held just before the first spring lockdown), and 2 of these students required the 25% “Extra time” for reading. A total of 6 students passed the VOSCE with no significant issues regarding delivery via the virtual platforms. There were no concerns in regards to exam metrics in any of the stations, and all stations contributed to the final analysis.

## Discussion

### Assessment unit

Invigilators were instrumental in the exam coordination and academics led various training sessions to ensure that the entire team understood the plan. Communication was crucial for the smooth running of the process, and there were two separate WhatsApp (telephone text channels) set up – one for all staff including examiners/patients and one for only the Assessment Unit staff to co-ordinate accurate timings. This became essential when, for example, one candidate took longer than anticipated to email a photograph of their completed prescription. One senior member of the assessment team had oversight on the day and was free of invigilation, understood the OSCE plan and process and could troubleshoot. Their role was central.

An overall spreadsheet ensured there were no overlaps between students. The master spreadsheet (Table 1) correlated with each invigilator script (Table 2). On the day, the changeover time of 2 min between stations was increased to 3 min and that meant ‘extra time’ students or stations requiring extra time to transfer document information could be accommodated.

Several security issues had to be considered. In addition to the usual initial student ID check, there was a specific room check, that involved a room sweep using the candidates webcam and phone check that asked the students to switch their phone on flight mode and place it on the floor. A security sticker had been attached to posted documents to ensure the student did not open the envelopes with exam materials before the exam, and the students were asked to demonstrate the intact envelope and security seal before opening it just before the start of the relevant station.

It was anticipated that there would be possible platform access issues for actors and examiners without access to university email accounts. This was overcome with separate links that were sent to the actors and examiners on the day of the exam. Running the pilot and the role player training were both crucial in identifying these challenges and trialing different approaches.

### Examiners

Examiners were all medical school clinical academics who had previous experience of examining in OSCEs. The examiners all undertook a training session (that was recorded if they could not attend ‘in person’) that outlined the format of the exam. They also received the exam station information and marking criteria the night before the exam so they could fully familiarise themselves with the station and paperwork given the novel format. In the immediate post VOSCE feedback session, they fed back that these interventions had been helpful to ensure smooth running on the day of the VOSCE, aswell as ongoing support during the exam via the Whatsapp group. One examiner fed back at the post OSCE meeting that they had struggled to mark a particular aspect of their station (where students had to verbally run through a practical procedure without the presence of any visual prompts) and this information fed into the group discussion around standard setting for that question.

### Students

The students were informed throughout the planning and were all involved in the piloting of this novel assessment. Challenges, proposed solutions and justifications were shared with the candidates, and their feedback and engagement was exceptionally useful. After the exam, two students flagged immediate concerns about some connectivity issues during the exam, and were reassured these issues had been reported by invigilators and would not be held against them in any marking. Several students also commented positively upon the strong preparation and support they had been given by the Assessment Unit and academics responsible for delivering the mock exam.

### Simulated patients

The simulated patients had all taken part in OSCEs before and commented that familiarity with the station at this novel stage of the VOSCE was helpful, although going forward would not necessarily be required. They were grateful for the training session that addressed any connectivity issues and practice ‘entering’ and ‘exiting’ the individual student ‘rooms’.

### Reflections

It was recognised by all those involved that the VOSCE process was labour intensive in both preparation and delivery. It became clear as the assessment ran that the preparation and large number of people involved were essential to maintain the quality of the assessment. Scaling this up to a full cohort assessment would not be achievable.

It was recognised that the platforms used on the day had performed optimally; had the platform not been stable for all parties for whatever reason on that day then the VOSCE may have had to be abandoned. A back-up platform for all parties had been identified, but the invitation set-up for a new platform would have taken time and further organisation.

In the design of a novel format of assessment, it is crucial at all steps to consider validity. Kane (2013) states that to validate use of test scores is to evaluate the plausibility of the claims based on the scores [11], with Cook and colleagues (2015) using Kane’s four inferences of Scoring, Generalisation, Implication and Extrapolation to provide a framework for validity [12]. Although we did not specifically use this framework to validate our VOSCE, Kane’s inferences can be clearly applied to our undertaking (See Table 3).

**Table 3:** Inferences of testing validity [12] applied to VOSCE

Inference	Description^a^	Application to VOSCE
Scoring	translating an observation into one or more scores	Use of marking checklists
Generalisation	using the score [s] as a reflection of performance in a test setting	Scores reflected the knowledge and performance, not the virtual technical skills
Implication	applying the score [s] to inform a decision or action	Standard setting to set the pass-mark
Extrapolation	using the score [s] as a reflection of real-world performance	Standard setting using the anchor statement of minimally competent Foundation Year 1 doctor

^a^descriptions derived from [12]

Finally, it was noted by the external examiners that some of the practical procedure stations, primarily those that involved talking through a scenario or directing a patient to undertake a clinical skill, only reached the ‘knows how’ level of Miller’s pyramid [13]; however apart from sending equipment to candidates remotely (which has its own logistical issues), there was no consensus how to address this and it was suggested that if this mode of assessment was to be adopted more widely, then a workplace based assessment process may be considered in parallel to assess this type of skill.

## Conclusion

Despite the pandemic, we were keen that students, who would not have been able to graduate otherwise, were given the opportunity to do so, and as such join the workforce at a crucial time. The marking structure was designed so that those students not passing the VOSCE did so because of their knowledge and/or performance not reaching the standard, and not due to the novel exam format or their online technical skills.

This novel format required creativity and innovation with both assessment and technology, whilst being continually mindful of assessment validity. Key lessons learnt were the importance of: keeping the purpose of the assessment in mind throughout any process of change; maintaining excellent engagement with all stakeholders, particularly the students; piloting and trialling options repeatedly; and the paradoxical flexibility that a near-obsessive degree of planning and back-up planning can give.

Finally, the process has highlighted the argument to move away from single high-stakes assessments towards more longitudinal programmes of assessment, which may be less affected by unforeseen major events. This has triggered a review of clinical assessments at our institution.

## Data Availability

not applicable.

## Abbreviations

VOSCE: Virtual Objective Structured Clinical Examination
OSCE: Objective Structured Clinical Examination
BRM: Borderline Regression Method
FY1: Foundation Year 1 doctor
